# Study on Absorption, Distribution and Excretion of a New Candidate Compound XYY-CP1106 against Alzheimer’s Disease in Rats by LC-MS/MS

**DOI:** 10.3390/molecules28052377

**Published:** 2023-03-04

**Authors:** Zili Guo, Bianbian Gao, Miaoliang Fan, Lisha Chen, Changjun Zhang, Xianrui Liang, Weike Su, Yuanyuan Xie

**Affiliations:** 1 College of Pharmaceutical Sciences, Key Laboratory of Pharmaceutical Engineering of Zhejiang Province, Collaborative Innovation Center of Yangtze River Delta Region Green Pharmaceuticals, Zhejiang University of Technology, Hangzhou 310014, China; 2 Interdisciplinary Research Academy, Zhejiang Shuren University, Hangzhou 310015, China

**Keywords:** XYY-CP1106, Alzheimer’s disease, LC-MS/MS, pharmacokinetics, tissue distribution, excretion study

## Abstract

XYY-CP1106, a candidate compound synthesized from a hybrid of hydroxypyridinone and coumarin, has been shown to be remarkably effective in treating Alzheimer’s disease. A simple, rapid and accurate high-performance liquid chromatography coupled with the triple quadrupole mass spectrometer (LC-MS/MS) method was established in this study to elucidate the pharmacokinetics of XYY-CP1106 after oral and intravenous administration in rats. XYY-CP1106 was shown to be rapidly absorbed into the blood (*T*_max_, 0.57–0.93 h) and then eliminated slowly (*T*_1/2_, 8.26–10.06 h). Oral bioavailability of XYY-CP1106 was (10.70 ± 1.72)%. XYY-CP1106 could pass through the blood–brain barrier with a high content of (500.52 ± 260.12) ng/g at 2 h in brain tissue. The excretion results showed that XYY-CP1106 was mainly excreted through feces, with an average total excretion rate of (31.14 ± 0.05)% in 72 h. In conclusion, the absorption, distribution and excretion of XYY-CP1106 in rats provided a theoretical basis for subsequent preclinical studies.

## 1. Introduction

Alzheimer’s disease (AD) is a degenerative disease of the central nervous system that occurs in old and pre-old age. It is characterized by cognitive and behavioral dysfunction [1]. The clinical manifestations include memory impairment, persistent intellectual decline, aphasia, loss of judgment ability and dyskinesia [2]. It is the third largest disease after cardiovascular diseases and tumors that disables and kills the elderly [3]. The main pathological feature of AD is the gradual loss of central nervous system function [4]. Although AD was discovered more than 100 years ago and related studies in the fields of neurology, pathology, molecular genetics, immunochemistry and computer modeling continue to develop, the pathogenesis and pathogenicity of AD remains ambiguous [5]. The pathogenesis of AD is complex, involving β-amyloid protein deposition, neurotransmitter deficiency, metal metabolism imbalance, overexpression of monoamine oxidase B (MAO-B), neuroinflammation, oxidative stress, tau protein hyperphosphorylation, etc. [6,7]. At present, acetylcholinesterase (AChE) inhibitors, N-methyl-D-aspartate (NMDA) receptor antagonists or a combination of them are used clinically to increase the content of neurotransmitters in the synaptic space to alleviate cognitive and behavioral disorders [8]. Until now, there have been five anti-AD drugs approved by the FDA, namely, the AChE inhibitor tacrine [9], donepezil [10], rivastigmine [11], galantamine [12] and the NMDA receptor antagonist memantine [13]. However, these single-target drugs have poor efficacy, which can only alleviate or improve the symptoms of AD patients to a certain extent but cannot prevent or reverse deterioration of the disease [14]. In view of the defects of traditional single-target drug therapy methods, the research and development of anti-AD drugs at home and abroad has developed towards the direction of multi-target drugs, that is, a multi-target ligands (MTDLs) strategy based on “one molecule, multiple targets”, which improves the therapeutic effect by simultaneously regulating multiple signal pathways in the pathogenesis of AD [15]. Compared with single-target drugs, multi-target drugs can make up for the low bioavailability of a single drug, the poor efficacy and insufficient patient tolerance and compliance caused by drug combination, and the toxicity of potential drug metabolism interactions, with unique potential [16,17]. In view of the importance of MAO-B, bio-metals and oxidative stress in the treatment of AD, our group designed and synthesized a series of new hydroxypyridinone–coumarin hybrids as multimodal MAO-B inhibitors and iron chelates to combat AD.

According to the pathogenesis of biological iron metabolism disorder and monoamine oxidase B (MAO-B) overexpression in the brain of AD patients, a series of dual-target anti-AD compounds were designed and synthesized by our research team in the early stage, and the candidate compound with the best activity XYY-CP1106 was obtained [18]. XYY-CP1106 was designed based on the common key nodes in the AD pathological signal pathway. In addition, the structure-based computer-aided drug design method was adopted, as well as the parallel principle, pharmacophore fusion and other drug design strategies. Theoretically, XYY-CP1106 could regulate the homeostasis of bio-iron, inhibit neuroinflammation and simultaneously reverse neurotoxicity and antioxidation. Among them, through the water maze experiment of in vitro pharmacological research, the patented new molecule XYY-CP1106 showed good protective effects on memory and cognition. In terms of drug activity screening, the experiments including iron ion chelating ability, MAO-B inhibitory activity and the mouse water maze experiment were investigated. The experimental results showed that XYY-CP1106 had high selectivity and strong chelating ability for Fe^3+^ (pFe^3+^ was 18.04), and its inhibitory activity for MAO-B was excellent (IC_50_ was 14.7 nM). In the scopolamine-induced AD mouse model, intraperitoneal injection of XYY-CP1106 (10 mg/kg) could significantly alleviate the behavioral and cognitive impairment of mice, reduce the latency of water maze navigation and increase the number of times the model mice pass the stage. Therefore, this study explored the pharmacokinetics, tissue distribution and excretory study of XYY-CP1106, which laid a solid foundation for further study [19]. Exploring the pharmacokinetic properties of novel drug candidates can provide a comprehensive understanding of the disposition of the drug in vivo, including the absorption, tissue distribution, metabolism and excretion [20].

**Figure 1:**
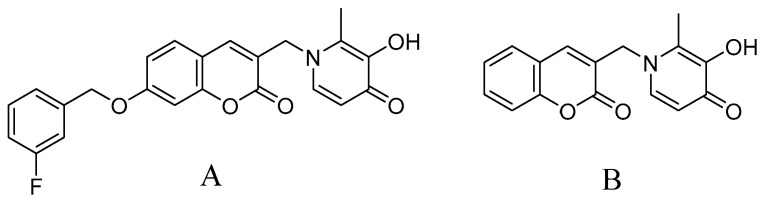
Chemical structures of XYY-CP1106 (**A**) and IS (**B**).

As XYY-CP1106 is a novel candidate compound (Figure 1A), it is necessary to study the pharmacokinetics of XYY-CP1106. In view of the low detection sensitivity of XYY-CP1106 in a biological matrix using traditional high-performance liquid chromatography (HPLC), a LC-MS/MS method used for the determination of XYY-CP1106 in rat plasma, tissues, feces and urine was developed. The absorption of XYY-CP1106 in rats was evaluated by oral administration and tail vein injection. Meanwhile, the tissue distribution and excretion characteristics of XYY-CP1106 in rats by oral administration were also studied.

## 2. Results

### 2.1. Method Validation

#### 2.1.1. Selectivity

**Figure 2:**
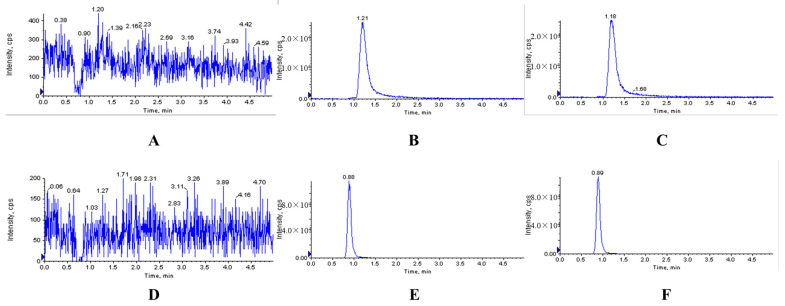
Representative MRM chromatograms of XYY-CP1106 and IS in rat plasma. (**A**) Blank plasma sample; (**B**) blank plasma spiked with XYY-CP1106; (**C**) actual plasma sample collected after oral administration of XYY-CP1106; (**D**) blank plasma sample; (**E**) blank plasma spiked with IS; (**F**) actual plasma sample collected after oral administration of IS.

**Figure 3:**
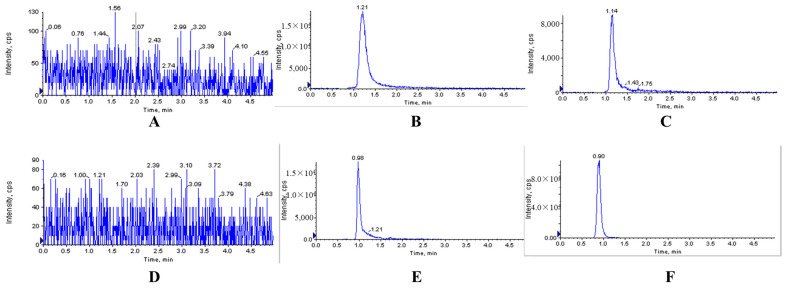
Representative MRM chromatograms of XYY-CP1106 and IS in brain tissue. (**A**) Blank brain tissue sample; (**B**) blank brain tissue sample spiked with XYY-CP1106; (**C**) actual brain tissue sample collected after oral administration of XYY-CP1106; (**D**) blank brain tissue sample; (**E**) blank brain tissue sample spiked with IS; (**F**) actual brain tissue sample collected after oral administration of IS.

**Figure 4:**
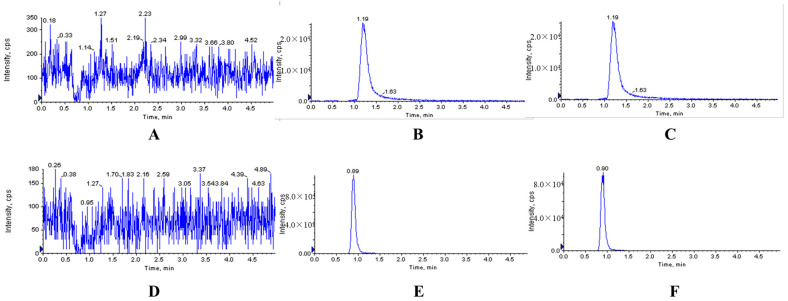
Representative MRM chromatograms of XYY-CP1106 and IS in rat feces. (**A**) Blank fecal sample; (**B**) blank fecal sample spiked with XYY-CP1106; (**C**) actual fecal sample collected after oral administration of XYY-CP1106; (**D**) blank fecal sample; (**E**) blank fecal sample spiked with IS; (**F**) actual fecal sample collected after oral administration of IS.

The typical chromatograms of blank and analyte-spiked plasma, tissues, feces and urine samples clearly showed that the optimized chromatographic method and extraction procedure produced clean sample extracts. There were no endogenous interferences at the retention time (RT) of XYY-CP1106 (1.21 min) or IS (0.89 min), shown in Figure 2, Figure 3 and Figure 4.

#### 2.1.2. Linearity and Sensitivity

The standard curve of XYY-CP1106 in plasma showed good linearity within the concentration range of 5–200 ng/mL, and the correlation coefficient (*r^2^*) was 0.9991. Meanwhile, the calibration curve in tissue homogenate, feces and urine also showed good linearities within the concentration range of 5–1000 ng/mL, with correlation coefficients *r^2^* > 0.9910. The LLOQ of XYY-CP1106 was 5 ng/mL in plasma, tissues, feces and urine, indicating that the established method had good sensitivity. The sensitivity of this method was sufficient to generate the plasma concentration–time curve of XYY-CP1106 after oral and tail vein administration in rats. The data were shown in Table 1.

#### 2.1.3. Precision and Accuracy

The intra-day and inter-day precision and accuracy of XYY-CP1106 in plasma, tissue homogenate, feces and urine were all within the acceptable range in LLOQ, LQC, MQC and HQC. As shown in Table 2, RSD (%) values of QC samples corresponding to low, medium and high concentrations in LLOQ and various matrices of rats were between 0.20% and 11.71%, and RE (%) values were between 92.84% and 105.20%. Therefore, the developed method was accurate to quantify XYY-CP1106 in rat plasma and different tissue matrices within a given concentration range.

#### 2.1.4. Extraction Recovery and Matrix Effect

The matrix effect ranges of XYY-CP1106 quality control samples from plasma, tis-sues, feces and the urine of six different rats was 94.48% to 104.49%, and the RSD (%) value was <8.21%, as shown in Table 3. These results indicated that the method was not affected by matrix composition.

#### 2.1.5. Stability

As shown in Table 4, XYY-CP1106 was stable in plasma, tissues, feces and urine under various storage conditions (automatic sampler for 24 h; freeze–thaw from −20 °C to room temperature three times or stored at −20 °C for the last 30 days), and RSD (%) values were all within the acceptable criteria. The deviation between the measured concentration and the test quality control level was less than 15% of its nominal concentration value.

### 2.2. Pharmacokinetic Analysis and Bioavailability

**Figure 5:**
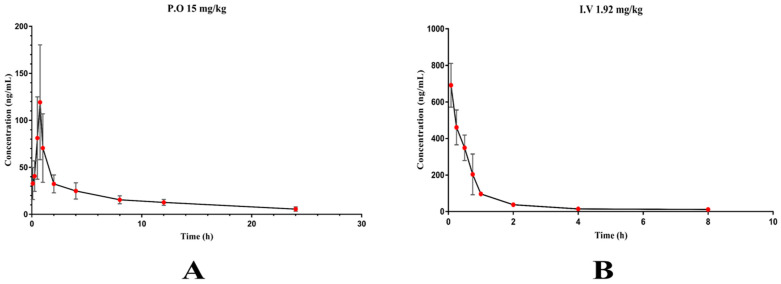
Plasma concentration–time curve of XYY-CP1106. (**A**) 15 mg/kg by intragastric administration; (**B**) 1.92 mg/kg by tail vein injection.

PK Solver plug-in was used to estimate the major pharmacokinetic parameters. A plot of the mean plasma concentration–time profile and the corresponding pharmacokinetic parameters were displayed in Table 5 and Figure 5. After oral administration of XYY-CP1106 to SD rats at doses of 15 mg/kg, *T*_max_ of rats was (0.75 ± 0.18) h, indicating that XYY-CP1106 could be absorbed into the blood quickly. There was a good absorption of XYY-CP1106 with the AUC_0-∞_ value of 502.93 ± 77.72 ng/mL×h. *T*_1/2_ was (9.16 ± 0.90) h, indicating that XYY-CP1106 was eliminated slowly. XYY-CP1106 exhibited good oral bioavailability with an absolute bioavailability (F) of (10.70 ± 1.72)% after a single oral administration of XYY-CP1106 to rats.

### 2.3. Tissue Distribution Study

**Figure 6:**
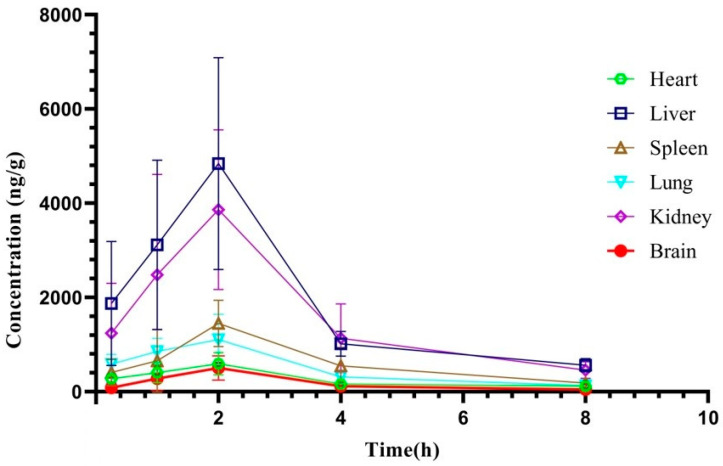
Tissue distribution of XYY-CP1106 in rats.

According to the results of pharmacokinetic study, the times at 0.25, 1, 2, 4 and 8 h were selected for tissue distribution study. The tissue distribution results of XYY-CP1106 in rats after oral administration (15 mg/kg) are shown in Figure 6. The results revealed that XYY-CP1106 was rapidly and widely distributed into all six tissues. As shown in Figure 5, XYY-CP1106 was mainly distributed in the liver and kidneys due to the higher concentration in the liver and kidneys than that in other tissues. Meanwhile, it was found that the main metabolic organ of XYY-CP1106 was the liver by comparing the concentration changes of XYY-CP1106 at 2 h and 4 h. After 2 h of oral administration, the concentrations of XYY-CP1106 in the brain of rats were (500.52 ± 260.12) ng/g, indicating that XYY-CP1106 could penetrate the blood–brain barrier (BBB).

### 2.4. Excretory Study

**Figure 7:**
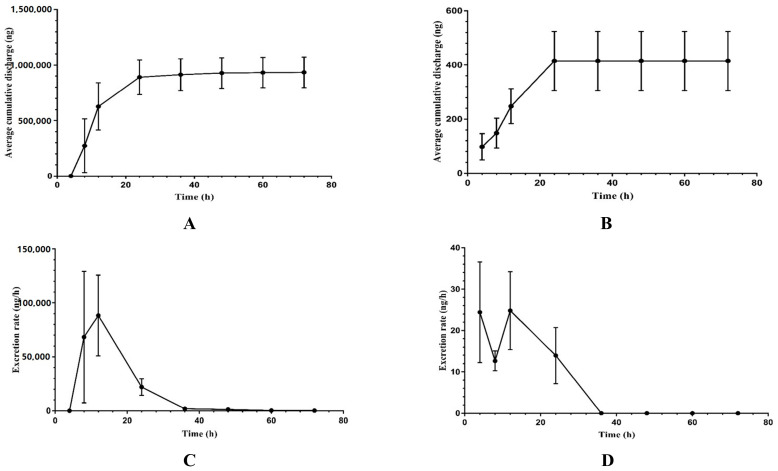
The excretion of XYY-CP1106 in rats. (**A**) The average cumulative excretion in feces; (**B**) the average cumulative excretion in urine; (**C**) the rate of excretion in feces; (**D**) the rate of excretion in urine.

The urinary and fecal cumulative excretion rates of XYY-CP1106 in rats after oral administration were shown in Figure 7. After rats were given 15 mg/kg XYY-CP1106 by gavage, their urine reached the maximum excretion rate at 4–12 h and feces at 8–12 h. The accumulated amount of XYY-CP1106 in urine did not increase after 24 h. Within 24 h, XYY-CP1106 excreted (0.01 ± 0.00)% of the dose through urine. In feces, XYY-CP1106 excreted (31.14 ± 0.05)% of the dose through feces within 72 h. It indicated that XYY-CP1106 was excreted mainly through feces.

## 3. Discussion

A stable, high-throughput, accurate and sensitive method is necessary and important for the study of absorption, tissue distribution and excretion [21]. In this study, the LC-MS/MS method based on triple quadrupole mass spectrometry was developed [22]. Firstly, the mass spectrum conditions of XYY-CP1106 and IS were optimized, such as the ion mode and the parameters including Declustering Potential, Collision Energy, Ion Source Gas 1, Ion Source Gas 2, Ion Spray Voltage, temperature, etc. [23] Then, the elution effects of different mobile phases and the addition of different concentrations of formic acid to the mobile phase were compared. Next, 0.2% formic acid water and 0.3% formic acid methanol with a ratio of 3:7 was selected as the final mobile phase [24]. Methodological investigation was carried out according to the methodological validation of biological matrix samples, and good specificity, good linear relationship (*r*^2^ > 0.99), high accuracy and precision were obtained. The matrix effect was 85–115%, all in line with the requirements [25].

XYY-CP1106 had high fat solubility due to a coumarin core [26]. Its low water solubility may be one of the reasons affecting its bioavailability [27]. The maximum plasma concentration of XYY-CP1106 was reached in 45 min after oral administration, and the maximum concentration was (153.48 ± 15.93) ng/mL, indicating that the absorption rate of the compound was fast [28,29,30]. AUC_0–24_ was (427.95 ± 66.09) ng/mL·h and AUC_0-∞_ was (502.93 ± 77.72) ng/mL·h, indicating that there was a good absorption of XYY-CP1106 [31]. Its *T*_1/2_ was (9.16 ± 0.90) h, which was relatively long [32,33,34]. The structure of XYY-CP1106 had a high affinity for iron ions and MAO-A enzymes [18,35]. The content of iron ions and MAO-B in the liver was high [36], so the content of compounds detected in the liver was the highest. It had been reported that the distribution of coumarins will also be high in the liver and kidney [37]. XYY-CP1106 was detected in brain tissue, indicating that it could penetrate the blood–brain barrier [38,39]. The prototype drug investigated in this study was mainly excreted through feces, with an average cumulative excretion of (0.94 ± 0.14) mg and an average excretion rate of (31.14 ± 0.05)%. The content in the tissue was significantly higher than that in the plasma. It may have higher fat solubility and better combination with the tissue and higher combination with the protein in the tissue, which was related to the characteristics of the compound itself [40]. However, it may also accumulate in liver and kidney tissue, so we should pay special attention to whether there is toxicity in the subsequent toxicity study [41,42].

By revealing the absorption and distribution characteristics of XYY-CP1106 at different time points, we can provide a good understanding and reference for the pharmacological effects of XYY-CP1106 and its potential role in targeted research in future work [43].

## 4. Materials and Methods

### 4.1. Chemicals and Reagents

XYY-CP1106 (C_23_H_18_FNO_5_, purity > 98%) and IS (C_16_H_13_NO_4_, purity > 98%) (Figure 1) were synthesized in our laboratory. The structures were confirmed by ^1^H NMR, ^13^C NMR, ESI-MS and high-resolution mass spectrometry (HRMS). HPLC-grade methanol was supplied by Merck (Darmstadt, Germany). HPLC-grade formic acid was purchased from Shanghai Aladdin Bio-Chem Technology Co., Ltd. (Shanghai, China). Ultrapure water (18.2 MΩ) was purified using the Milli-Q^®^ IQ 7000 Purification System (Molsheim, France). Other reagents used in this experiment were all analytical-grade and were obtained from Yongda Chemical Reagent Company (Tianjin, China).

### 4.2. Animals

Specific pathogen-free (SPF)-grade male Sprague-Dawley (SD) rats (210 ± 30 g, 8 weeks) were provided by Zhejiang Academy of Medical Sciences (Hangzhou, China). The rats were kept in an animal room with an ambient temperature of 22 ± 2 °C, a relative humidity of 55 ± 5% with 12 h light/dark cycles and were observed for one week in the Experimental Animal Center of the Zhejiang Province (Hangzhou, China) before starting the experiments. Before the experiment, the rats fasted from standard laboratory food for 12 h but were free to drink water. The experimental protocols involving animals were strictly followed from the Guide for the Institutional Animal Care and Use Committee of Zhejiang University of Technology Laboratory Animal Center (20190301036 and 20220411038).

### 4.3. Instrumentation and Analytical Conditions

The chromatographic separation experiment was carried out on a 1260 HPLC system (Agilent, CA, USA) equipped with a Triple Quad 4500 system (SCIEX, Bremen, Germany) using composite electrospray ionization (ESI) in positive ion mode. The separation was operated on a BEH C18 column (50 × 2.1 mm, 1.8 µm, Waters, Milford, MA, USA) and the column temperature was maintained at 35 °C. The samples were eluted using an isocratic elution of 30% A (0.2% formic acid aqueous solution) and 70% B (0.3% formic acid methanol) at a flow rate of 0.2 mL/min. Total run time for each sample was 5 min. The volume of the injection solution was 5 μL.

The mass spectrometer was operated in positive ion mode with selected multiple reaction monitoring (MRM) mode for all the analytes. The precursor-to-product ion pairs used for XYY-CP1106 and IS were *m/z* 407.9→282.9/109.0 (DP 120 eV, CE 58 eV) and *m/z* 283.9→159.0/115.1 (DP 120 eV, CE 30 eV). The optimized MS parameters were set as follows: collision gas (CAD) at 9 psi, curtain gas (CUR) at 30 psi, nebulizer gas (GS1) at 45 psi, heated by N_2_ gas (GS2) at 40 psi, Ion Spray Voltage at 5500 V and temperature at 500 °C.

**Figure 8:**
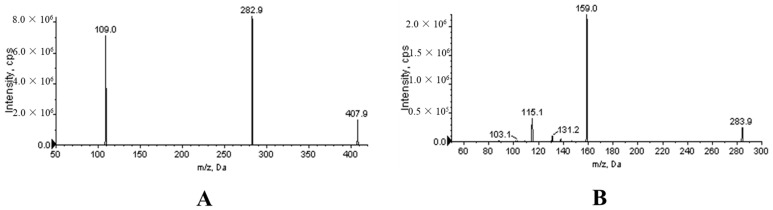
Product ion mass spectra of XYY-CP1106 (**A**) and IS (**B**).

The ion scanning mass spectrograms of the products of XYY-CP1106 and IS were shown in Figure 8. Non-compartmental calculations were performed using the PK Solver plug-in (China Pharmaceutical University, Nanjing, China) to estimate the main pharmacokinetic parameters [44]. All values were expressed as the mean ± standard deviation.

### 4.4. Preparation of Calibration Standards and Quality Control Samples

#### 4.4.1. Plasma Pharmacokinetic Study

XYY-CP1106 and IS stock solution (10 mL) with a concentration of 1.0 mg/mL in methanol were prepared; then, they were stored in a refrigerator at 4 °C before use. Next, the working solution was prepared for the determination of the standard curve. XYY-CP1106 stock solution was gradually diluted by methanol to the concentrations of 50, 100, 250, 500, 1000, 1600 and 2000 ng/mL as the working solution of the standard curve (calibration standard, CS). Then, it was diluted to 50, 100, 1000 and 1600 ng/mL as the working solution of quality control (QC). IS stock solution was diluted to 1000 ng/mL as working solution. The CS was prepared by spiking the appropriate amount of XYY-CP1106 and IS working solutions into drug-free rat plasma of 100 μL to make XYY-CP1106 at the final concentrations of 5, 10, 25, 50, 100, 160 and 200 ng/mL and IS at the final concentration of 100 ng/mL. To assess the accuracy and precision of the analysis method, QC samples were prepared at four concentration levels: 5 ng/mL (lower limit of quantification, LLOQ), 10 ng/mL (low QC, LQC), 50 ng/mL (medium QC, MQC) and 160 ng/mL (high QC, HQC).

#### 4.4.2. Tissue Distribution Study

The stock solutions were the same as those used in pharmacokinetic studies. The XYY-CP1106 stock solution was serially diluted with methanol to prepare working solutions of XYY-CP1106 at final concentrations of 50, 100, 500, 1000, 5000, 8000 and 10,000 ng/mL, and the IS stock solution was diluted by methanol into 1000 ng/mL. In order to obtain XYY-CP1106 CS samples (5, 10, 50, 100, 500, 800 and 1000 ng/mL), 100 μL of XYY-CP1106 working solution with various gradients and 1000 ng/mL of IS were successively added to 200 μL of blank tissue homogenate; finally, ice methanol was added for protein precipitation. The same method was used to prepare QC samples with LLQC, LQC, MQC and HQC (5, 10, 100 and 800 ng/mL).

#### 4.4.3. Excretion Study

The stock solution and working solution were the same as those used in the tissue distribution study.

### 4.5. Sample Preparation

#### 4.5.1. Preparation of Plasma Samples

Amounts of 100 μL plasma, 100 μL IS working solution (1000 ng/mL), 100 μL aqueous solution containing 0.2% formic acid and 700 μL ice methanol for protein precipitation were successively added into the 1.5 mL centrifuge tube, and it was vortexed for 30 s. Then, it was centrifuged at 13,000 rpm for 10 min at 4 °C, and the upper clarifying solution was extracted and placed in a new 1.5 mL tube and was centrifuged again under the same conditions. Five microliters of the sample were measured and injected into the LC-MS/MS system for analysis.

#### 4.5.2. Preparation of Tissue Samples

The tissue sample was weighed and cut into pieces. Normal saline was added to brain tissue at the ration of 1:1 (g: mL) and other tissues at the ration of 1:3 (g: mL). The tissue homogenate was symmetrically placed in a tissue homogenizer to obtain tissue homogenate.

Amount of 0.5 mL of tissue homogenate and 1.5 mL of acetonitrile were added into a 5 mL centrifuge tube for protein precipitation by vortex, and 1 mL of vortex solution was added into a 1.5 mL centrifuge tube and centrifuged at 13,000 revolutions at for 10 min 4 °C. Then, 900 μL supernatant and 100 μL IS working solution were added into a 1.5 mL centrifuge tube to vortex for 30 s. It was centrifuged twice under the same conditions; then, the supernatant (5 μL) was injected into the LC-MS/MS system.

#### 4.5.3. Preparation of Fecal Samples

After the collected feces were dried at room temperature, a pestle and mortar were used to uniformly crush the feces collected at each time slot. Then, 0.2 g of ground feces were accurately weighed into a 5.0 mL centrifuge tube, 4 mL of methanol solution was added and then it was put into the ultrasonic instrument for ultrasonic extraction for 4 h. Then, 100 μL of feces samples were added into a 1.5 mL centrifuge tube. Next, it was treated the same as the plasma samples.

#### 4.5.4. Preparation of Urine Samples

Urine samples were collected and processed directly. An aliquot of 100 µL of urine sample was taken and processed in the same manner as the plasma samples.

### 4.6. Method Validation

The analytical method was validated according to the bioanalytical method guidelines suggested by the Chinese *Pharmacopoeia of the People’s Republic of China* (2020 Edition) [45]. The specificity, linearity, accuracy, precision, extraction recovery, matrix effect and stability of the LC-MS/MS method established by XYY-CP1106 in rat plasma, heart, liver, spleen, lung, kidney and brain tissues and urine and fecal biological matrices were verified.

#### 4.6.1. Specificity

To ensure no endogenous biological matrix components interfered with the determination, the specificity of the method was evaluated by comparing the chromatograms of blank rat matrices with those obtained from the corresponding matrices spiked with XYY-CP1106 and IS working solutions and actual biological samples.

#### 4.6.2. Linearity

The preparation of the standard curves of plasma, tissue and excretion were shown in Section 4.5.1, Section 4.5.2 and Section 4.5.3, respectively. Standard calibration curves were constructed by plotting the measured pear area ratios of XYY-CP1106 to IS versus the concentration of XYY-CP1106 (*n* = 3).

#### 4.6.3. Accuracy and Precision

Accuracy is expressed as relative error (RE) and precision is expressed as RSD. The intra-day precision of the method was evaluated by analyzing QC at four different concentration levels (LLOQ-QC, LQC, MQC and HQC, *n* = 6) and it was repeated in three consecutive days for inter-day precision (*n* = 18). The accuracy of the method was evaluated by analyzing the QC sample within one day and adding the standard with known concentration to the QC sample (*n* = 6). The acceptable limit of RSD and CE was ±15% of the nominal value (lower limit of quantification and low concentration were not more than 20%).

#### 4.6.4. Extraction Recovery and Matrix Effect

The recovery rate of analytes in the analysis was determined by comparing the average peak area obtained by adding the extracted plasma, tissue and excretory samples into the standard with the peak area obtained by directly injecting the standard into the biological matrix and then extracting the XYY-CP1106 of different concentrations (LQC, MQC and HQC) (*n* = 6). The extraction recovery rate of the 6 batches should be between 85 and 115%, and the low concentration should be between 80 and 120%.

For each matrix, we calculated the matrix factor of each analyte and IS by calculating the peak area in the presence of the matrix (measured by adding analytes and IS after extraction from the blank matrix) and the ratio of the corresponding peak area (pure solution of analyte and IS) without matrix. Further, the matrix factor normalized by IS was calculated by dividing the matrix factor of the analyte by the matrix factor of IS. The coefficient of variation of the IS normalization matrix factors calculated from six batches of matrices should not be greater than 15% (low concentration should not be greater than 20%). The determination was carried out at low, medium and high concentrations, respectively.

#### 4.6.5. Stability

The stability of XYY-CP1106 standard in plasma, tissues, feces and urine biological samples under different storage conditions was analyzed, including the stability of samples stored at room temperature for 24 h in an automatic sampler, the freeze–thaw stability after three cycles (−20 °C to room temperature was defined as a cycle), and −20 °C for the last 30 days. When the deviation from the standard value was within 15% (low concentration was within 20%), the sample was considered stable. The concentration of standard substance in plasma was 10 and 160 ng/mL. The standard concentration in tissues, feces and urine was 10 and 800 ng/mL.

### 4.7. Pharmacokinetic Study

#### 4.7.1. Preparation of Drug Solution

XYY-CP1106 was almost insoluble in water due to its high lipid solubility. A certain amount of XYY-CP1106 was weighed and dissolved completely in methanol. β-cyclodextrin aqueous solution was added to make it 50% methanol and 30% β-cyclodextrin solution. Then, it was placed in ultrasound for 4 h under an ultrasonic bath after vortex. Next, it was freeze-dried for 2 days; then, it was redissolved in PBS solution to the final concentration of 1.0 mg/mL.

#### 4.7.2. Plasma Pharmacokinetic Study

For oral administration, six SD rats were fed with freely available food and water and fasted with free access to water for 12 h before drug administration. The rats in each group were weighed and recorded; then, the oral dose was 15 mg/kg. The average weight of rats was 200 ± 30 g. At 0.083, 0.25, 0.5, 0.75, 1, 2, 4, 6, 8, 12 and 24 h after oral administration, 0.3 mL of blood samples were collected from the suborbital vein and put into a 1.5 mL heparin sodium tube. The collected plasma sample was centrifuged at 4 °C for 10 min at 3500 revolutions, and 100 μL of supernatant was taken and stored at −20 °C for future use.

For tail vein injection, six SD rats were weighed and recorded; then, XYY-CP1106 (0.384 mg/mL, 1.92 mg/kg) filtered by a 0.22 µm filter membrane was administered via tail vein injection. An amount of 0.3 mL of blood samples were collected from the suborbital vein into heparin sodium tubes at 0.083, 0.25, 0.5, 0.75, 1, 2, 4, 6, 8, 12 and 24 h after caudal intravenous administration. The collected plasma samples were treated in the same way as oral administration.

The oral absolute bioavailability (F) in rats was calculated according to the following equation:

F = (AUC_oral(0–∞)_ × Dose_iv_)/(AUC_iv(0–∞)_ × Dose_oral_) × 100%, (1)

Notes: AUC_oral(0–∞)_ was the area under the curve of oral administration; AUC_iv(0–∞)_ was the area under the curve of tail vein injection; Dose_oral_ was the oral dose; Dose_iv_ was the tail vein injection dose.

#### 4.7.3. Tissue Distribution Study

Thirty SD rats were randomly divided into 5 groups (*n* = 6), fed with freely available food and water and fasted with free access to water for 12 h before drug administration. All rats were weighed and recorded. The 5 groups of rats were given a single oral dose (15 mg/kg) of XYY-CP1106 (1.0 mg/mL) and sacrificed with cervical dislocation at 0.25, 1, 2, 4 and 8 h, respectively. The heart, liver, spleen, lungs, kidneys and brain of the rats were quickly removed, and the blood was carefully rinsed off with physiological saline (0.9%). The normal saline on the surface of all tissue samples was dried with filter paper then stored in a refrigerator at −80 °C.

#### 4.7.4. Excretory Study

Ten rats were fed with freely available food and water and fasted with free access to water for 12 h before drug administration. They were given a single dose of XYY-CP1106 (15 mg/kg, 1.0 mg/mL) and then put into metabolic cages. Feces and urine were collected at 0–4, 4–8, 8–12, 12–24, 24–36, 36–48, 48–60, 60–72, 72–84 and 84–96 time slots, respectively. The feces collected at each time slot were air-dried to record their weight and stored in a refrigerator at −20 °C before use. The urine volume was recorded at each time slot and stored in a refrigerator at −20 °C before use.

## 5. Conclusions

This is the first report to evaluate the pharmacokinetic study of XYY-CP1106 in SD rats. A sensitive LC-MS/MS method was developed and validated for quantitative bioassay of XYY-CP1106 in plasma, tissues, feces and urine. The method was accurate and fast, only taking 5 min per sample. XYY-CP1106 was distributed rapidly and extensively after oral administration. XYY-CP1106 exhibited good oral bioavailability with an absolute bioavailability (F) of (10.70 ± 1.72)% after a single oral administration of XYY-CP1106 to rats. XYY-CP1106 had the highest content in brain tissue within 2 h, penetrating the BBB. The tissue distribution indicated that XYY-CP1106 may be a novel promising compound with targeted anti-AD properties. The results provided a theoretical basis for preclinical and clinical studies of XYY-CP1106.

## Figures and Tables

**Table 1:** Standard calibration curves and LLOQ of XYY-CP1106 in different matrices (*n* = 3).

Matrix	Equation	Range (ng/mL)	LLOQ (ng/mL)
Plasma	*y =* 0.018*x* + 0.006 (*r*^2^ = 0.9991)	5–200	5
Heart	*y* = 0.014*x* + 0.014 (*r*^2^ = 0.9986)	5–1000	5
Liver	*y* = 0.017*x* + 0.012 (*r*^2^ = 0.9997)	5–1000	5
Spleen	*y* = 0.016*x* + 0.003 (*r*^2^ = 0.9993)	5–1000	5
Lung	*y* = 0.014*x* − 0.004 (*r*^2^ = 0.9997)	5–1000	5
Kidney	*y* = 0.015*x* + 0.012 (*r*^2^ = 0.9997)	5–1000	5
Brain	*y* = 0.014*x* + 0.010 (*r*^2^ = 0.9997)	5–1000	5
Urine	*y* = 0.014*x* + 0.016 (*r*^2^ = 0.9910)	5–1000	5
Feces	*y* = 0.010*x* + 0.017 (*r*^2^ = 0.9991)	5–1000	5

**Table 2:** Intra-day and inter-day precision, accuracy of XYY-CP1106.

Matrix	Spiked (ng/mL)	Precision (RSD, %)		Accuracy (%) (*n* = 6)
		Intra-Day (*n* = 6)	Inter-Day (*n* = 18)	
Plasma	5	5.07	3.64	104.28
	10	1.21	2.90	102.97
	50	0.49	1.22	101.02
	160	0.20	0.95	102.87
Heart	5	7.67	8.48	98.10
	10	5.69	7.18	94.41
	100	8.15	7.73	97.21
	800	3.73	6.52	96.03
Liver	5	6.05	7.44	104.82
	10	7.93	7.49	105.2
	100	2.11	5.39	102.62
	800	4.31	5.51	98.51
Spleen	5	7.40	9.68	105.13
	10	7.56	7.22	105.20
	100	6.06	6.45	95.86
	800	5.76	5.32	92.84
Lung	5	8.91	7.34	101.26
	10	10.48	9.35	102.64
	100	5.74	4.64	102.19
	800	3.36	4.17	96.94
Kidney	5	7.82	11.62	98.10
	10	7.05	9.01	94.41
	100	7.56	6.12	97.21
	800	4.91	3.63	96.03
Brain	5	10.6	9.65	100.78
	10	5.54	7.11	104.69
	100	5.95	4.47	97.21
	800	4.85	4.42	95.41
Feces	5	6.32	8.31	102.15
	10	5.61	4.46	101.12
	100	4.77	4.12	99.88
	800	4.85	4.42	99.28
Urine	5	4.95	9.79	94.79
	10	11.71	8.97	101.10
	100	6.33	5.51	102.59
	800	3.34	3.30	102.41

**Table 3:** Extraction recovery and matrix effect of XYY-CP1106 (*n* = 6).

Matrix	Nominal Concentration (ng/mL)	Extraction Recovery (%, Mean ± SD)	Matrix Effect (%, Mean ± SD)
Plasma	10	99.35 ± 3.96	100.86 ± 6.30
	50	96.87 ± 3.54	97.54 ± 5.36
	160	100.46 ± 1.39	101.74 ± 2.51
Heart	10	109.91 ± 2.26	98.36 ± 7.52
	100	101.73 ± 6.38	95.70 ± 5.72
	800	108.08 ± 5.24	102.58 ± 5.94
Liver	10	108.07 ± 3.93	104.24 ± 7.71
	100	102.23 ± 5.91	98.77 ± 7.49
	800	100.78 ± 1.81	101.45 ± 6.33
Spleen	10	108.04 ± 3.71	104.32 ± 6.70
	100	106.63 ± 3.29	99.73 ± 7.54
	800	99.06 ± 6.10	102.91 ± 7.31
Lung	10	103.24 ± 3.69	101.01 ± 7.01
	100	98.48 ± 4.10	96.42 ± 6.99
	800	100.72 ± 3.93	100.70 ± 4.92
Kidney	10	104.75 ± 5.60	99.93 ± 6.65
	100	94.34 ± 3.61	99.31 ± 5.95
	800	102.33 ± 5.30	102.62 ± 6.16
Brain	10	106.15 ± 8.78	102.61 ± 4.27
	100	100.90 ± 5.46	99.36 ± 7.68
	800	98.91 ± 2.42	94.48 ± 4.40
Feces	10	97.99 ± 6.50	101.85 ± 6.02
	100	103.99 ± 2.20	100.19 ± 3.87
	800	101.44 ± 0.94	102.58 ± 6.21
Urine	10	106.49 ± 3.78	99.57 ± 8.17
	100	107.78 ± 5.96	98.84 ± 3.85
	800	105.40 ± 6.55	104.49 ± 6.57

**Table 4:** Stability of XYY-CP1106 in plasma, tissue, feces and urine (*n* = 6).

Matrix	Spiked (ng/mL)	24 h (Automatic Sampler)		Freeze–Thaw at −20 °C		Stored at −20 °C for 30 Days	
		Precision (%, RSD)	Accuracy (%)	Precision (%, RSD)	Accuracy (%)	Precision (%, RSD)	Accuracy (%)
Plasma	10	4.61	100.97	1.48	97.20	9.15	101.09
	160	0.49	99.92	1.70	99.79	5.02	97.03
Heart	10	7.15	107.09	5.24	95.63	5.85	94.72
	800	7.20	106.65	9.77	99.82	4.11	97.51
Liver	10	11.41	99.32	9.92	94.59	9.77	99.60
	800	7.45	96.3	7.35	99.63	4.56	100.98
Spleen	10	8.62	101.97	15.39	103.61	6.45	99.67
	800	8.55	92.34	7.14	97.54	2.51	98.02
Lung	10	15.86	98.59	9.88	97.42	6.06	98.89
	800	5.92	102.03	4.64	101.74	2.78	103.02
Kidney	10	14.83	103.91	18.22	99.06	7.44	98.10
	800	6.78	103.48	6.54	101.88	6.37	97.36
Brain	10	8.17	102.78	12.88	103.21	8.06	98.22
	800	4.54	97.04	3.95	95.94	6.62	94.08
Feces	10	6.51	99.33	7.90	100.5	6.44	94.98
	800	4.54	97.04	3.95	95.94	5.13	96.50
Urine	10	18.88	100.84	15.33	96.55	4.34	99.57
	800	4.72	100.47	4.62	98.70	3.93	100.46

**Table 5:** The main pharmacokinetic parameters of XYY-CP1106 (*n* = 6).

PK Parameters	I.V (1.92 mg/kg)	P.O (15 mg/kg)
AUC_0–24_ (ng/mL × h)	540.14 ± 43.71	427.95 ± 66.09
AUC_0–∞_ (ng/mL × h)	599.20 ± 31.78	502.93 ± 77.72
*T*_1/2_ (h)	3.50 ± 2.24	9.16 ± 0.90
CL (mL/h × kg)	1.61 ± 0.09	30.48 ± 5.28
V (mL/kg)	4.03 ± 1.80	401.70 ± 69.59
*C*_max_ (ng/mL)	691.20 ± 119.86	153.48 ± 15.93
*T*_max_ (h)	0.083 ± 0.00	0.75 ± 0.18
F (%)	/	10.70 ± 1.72

## Data Availability

The data that support the findings of this study are available from the corresponding author upon reasonable request.

## Sample Availability

Samples of the compounds (XYY-CP1106 and IS) are available from the authors.

## References

[1] Tagarelli A., Piro A., Tagarelli G., Lagonia P., Quattrone A. (2006). Alois Alzheimer: A hundred years after the discovery of the eponymous disorder. Int. J. Biomed. Sci..

[2] Lane C.A., Hardy J., Schott J.M. (2018). Alzheimer’s disease. Eur. J. Neurol..

[3] Soria Lopez J.A., Gonzalez H.M., Leger G.C. (2019). Alzheimer’s disease. Handb. Clin. Neurol..

[4] Mantzavinos V., Alexiou A. (2017). Biomarkers for Alzheimer’s disease diagnosis. Curr. Alzheimer Res..

[5] Oboudiyat C., Glazer H., Seifan A., Greer C., Isaacson R.S. (2013). Alzheimer’s Disease. Semin. Neurol..

[6] Weller J., Budson A. (2018). Current understanding of Alzheimer’s disease diagnosis and treatment. F1000Research.

[7] Savelieff M.G., Nam G., Kang J., Lee H.J., Lee M., Lim M.H. (2018). Development of multifunctional molecules as potential therapeutic candidates for Alzheimer’s disease, Parkinson’s disease, and Amyotrophic lateral sclerosis in the last decade. Chem. Rev..

[8] Briggs R., Kennelly S.P., O’Neill D. (2016). Drug treatments in Alzheimer’s disease. Clin. Med..

[9] Sameem B., Saeedi M., Mahdavi M., Shafiee A. (2017). A review on tacrine-based scaffolds as multi-target drugs (MTDLs) for Alzheimer’s disease. Eur. J. Med. Chem..

[10] Brewster J.T., Dell’Acqua S., Thach D.Q., Sessler J.L. (2019). Classics in Chemical Neuroscience: Donepezil. ACS Chem. Neurosci..

[11] Hroudová J., Singh N., Fišar Z., Ghosh K.K. (2016). Progress in drug development for Alzheimer’s disease: An overview in relation to mitochondrial energy metabolism. Eur. J. Med. Chem..

[12] Coyle J., Kershaw P. (2001). Galantamine, a cholinesterase inhibitor that allosterically modulates nicotinic receptors: Effects on the course of Alzheimer’s disease. Biol. Psychiatry.

[13] Parsons C.G., Danysz W., Dekundy A., Pulte I. (2013). Memantine and Cholinesterase Inhibitors: Complementary Mechanisms in the Treatment of Alzheimer’s Disease. Neurotox. Res..

[14] Bachurin S.O., Bovina E.V., Ustyugov A.A. (2017). Drugs in Clinical Trials for Alzheimer’s Disease: The Major Trends. Med. Res. Rev..

[15] Eratne D., Loi S.M., Farrand S., Kelso W., Velakoulis D., Looi J.C. (2018). Alzheimer’s disease paper 1: Clinical update on epidemiology, pathophysiology and diagnosis. Australas. Psychiatry.

[16] Anand R., Gill K.D., Mahdi A.A. (2014). Therapeutics of Alzheimer’s disease: Past, present and future. Neuropharmacology.

[17] Bondi M.W., Edmonds E.C., Salmon D.P. (2017). Alzheimer’s disease: Past, present, and future. J. Int. Neuropsychol. Soc..

[18] Zhang C.J., Yang K., Yu S., Su J., Yuan S.L., Han J.X., Chen Y., Gu J.P., Zhou T., Bai R.R. (2019). Design, synthesis and biological evaluation of hydroxypyridinone-coumarin hybrids as multimodal monoamine oxidase B inhibitors and iron chelates against Alzheimer’s disease. Eur. J. Med. Chem..

[19] Tan Y.F., Wang R.Q., Wang W.T., Wu Y., Ma N., Lu W.Y., Zhang Y., Zhang X.P. (2021). Study on the pharmacokinetics, tissue distribution and excretion of laurolitsine from Litsea glutinosa in Sprague-Dawley rats. Pharm. Biol..

[20] Fan A., Zhang Y.-L., Zhang Q., Wei J., Lu X., Ren G., Zhao D., Li N., Zhu H., Chen X. (2018). Evaluation of the pharmacokinetics, tissue distribution and excretion studies of YMR-65, a tubulin polymerization inhibitor with potential anticancer activity, in rats using UPLC-MS/MS. Xenobiotica.

[21] Taneja I., Raghuvanshi A., Raju K.S.R., Awasthi P., Rashid M., Singh S., Goel A., Singh S.P., Wahajuddin M. (2019). Bioavailability, tissue distribution and excretion studies of a potential anti-osteoporotic agent, medicarpin, in female rats using validated LC–MS/MS method. J. Pharm. Biomed. Anal..

[22] Wang F.G., Cao J., Hou X.Q., Li Z.Y., Qu X.L. (2018). Pharmacokinetics, tissue distribution, bioavailability and excretion of nuciferine, an alkaloid from lotus, in rats by LC-MS/MS. Drug Dev. Ind. Pharm..

[23] Zheng Q., Wang R., Zhang N., Wang C., Li P. (2020). In vivo pharmacokinetics, distribution, and excretion of an anticancer agent isolated from red ginseng, in rat. Xenobiotica.

[24] Lu J.J., Pan Q.Q., Zhou J.Q., Weng Y., Chen K.L., Shi L., Zhu G.X. (2022). Pharmacokinetics, distribution, and excretion of sodium oligomannate, a recently approved anti-Alzheimer’s disease drug in China. J. Pharm. Anal..

[25] Li B., Lu M., Chu Z.X., Lei S.S., Sun P.L., Xiong S., Chen S.H. (2019). Pharmacokinetics, bioavailability and urinary excretion of scopolin and its metabolite scopoletin in Sprague-Dawley rats by LC-MS/MS. Biomed. Chromatogr..

[26] Belubbi T., Shevade S., Dhawan V., Sridhar V., Majumdar A., Nunes R., Araújo F., Sarmento B., Nagarsenker K., Steiniger F. (2018). Lipid Architectonics for Superior Oral Bioavailability of Nelfinavir Mesylate: Comparative in vitro and in vivo Assessment. AAPS PharmSciTech.

[27] Hurst S., Loi C.M., Brodfuehrer J., El-Kattan A. (2007). Impact of physiological, physicochemical and biopharmaceutical factors in absorption and metabolism mechanisms on the drug oral bioavailability of rats and humans. Expert Opin. Drug Metab. Toxicol..

[28] Teruo M., Erik B., Nicholas B. (2021). Factors and dosage formulations affecting the solubility and bioavailability of P-glycoprotein substrate drugs. Expert Opin. Drug Metab. Toxicol..

[29] Abuhelwa A.Y., Williams D.B., Upton R.N., Foster D.J. (2017). Food, gastrointestinal pH, and models of oral drug absorption. Eur. J. Pharm. Biopharm..

[30] Chen X., Zhu P., Liu B., Wei L., Xu Y. (2018). Simultaneous determination of fourteen compounds of *Hedyotis diffusa* Willd extract in rats by UHPLC–MS/MS method: Application to pharmacokinetics and tissue distribution study. J. Pharm. Biomed. Anal..

[31] Hey J.A., Yu J.Y., Versavel M., Abushakra S., Kocis P., Power A., Kaplan P.L., Amedio J., Tolar M. (2018). Clinical pharmacokinetics and safety of ALZ-801, a novel prodrug of Tramiprosate in development for the treatment of Alzheimer’s disease. Clin. Pharmacokinet..

[32] Smith D.A., Beaumont K., Maurer T.S., Di L. (2018). Relevance of Half-Life in Drug Design. J. Med. Chem..

[33] Jones H.M., Tolsma J., Zhang Z., Jasper P., Luo H., Weber G.L., Wright K., Bard J., Bell R., Messing D. (2021). A physiologically-based pharmacokinetic model for the prediction of “Half-Life Extension” and “Catch and Release” monoclonal antibody pharmacokinetics. CPT Pharmacomet. Syst. Pharmacol..

[34] Bazzo G.C., Pezzini B.R., Stulzer H.K. (2020). Eutectic mixtures as an approach to enhance solubility, dissolution rate and oral bioavailability of poorly water-soluble drugs. Int. J. Pharm..

[35] Kozochkin D.A., Manukhina E.B., Downey H.F., Tseilikman O.B., Komelkova M.V., Vasilyeva M.V., Lapshin M.S., Sahabutdinov M.N., Lazuko S.S., Tseilikman V.E. (2017). The role of microsomal oxidation in the regulation of monoamine oxidase activity in the brain and liver of rats. Gen. Physiol. Biophys..

[36] Andrey V.K., Dmitry Y.Y., Yury A.V., Ofelja A.A. (1992). Intracellular free iron in liver tissue and liver homogenate: Studies with electron paramagnetic resonance on the formation of paramagnetic complexes with desferal and nitric oxide. Free Radic. Biol. Med..

[37] Zhang Y.B., Deng G.G., Wang T.X., Liu L., Yang X.W. (2019). Tissue distribution study of Angelica dahurica *cv.* Yubaizhi in rat by ultra-performance liquid chromatography with tandem mass spectrometry. J. Pharm. Biomed. Anal..

[38] Yang Y.-F., Zhang L., Yang X.-W. (2018). Distribution Assessments of Coumarins from Angelicae Pubescentis Radix in Rat Cerebrospinal Fluid and Brain by Liquid Chromatography Tandem Mass Spectrometry Analysis. Molecules.

[39] Yang Y.-F., Zhang Y.-B., Chen Z.-J., Zhang Y.-T., Yang X.-W. (2018). Plasma pharmacokinetics and cerebral nuclei distribution of major constituents of Psoraleae fructus in rats after oral administration. Phytomedicine.

[40] Ge Y., Chen S., Luo Q., Wang C.-P., Hao J., He J., Chen X., Yang X., Li J., Chang Y.-X. (2019). The Tissue Distribution of Four Major Coumarins after Oral Administration of Angelicae Pubescentis Radix Extract to Rats Using Ultra-High-Performance Liquid Chromatography. Evid. -Based Complement. Altern. Med..

[41] Hirono H., Watanabe K., Hasegawa K., Hiroyasu K., Shibasaki K., Ohkoshi S. (2018). Anti-Dementia Drugs and Hepatotoxicity–Report of Two Cases. Int. J. Gerontol..

[42] Patocka J., Jun D., Kuca K. (2008). Possible role of hydroxylated metabolites of tacrine in drug toxicity and therapy of Alzheimer’s disease. Curr. Drug Metab..

[43] Zeng X., Su W., Zheng Y., He Y., He Y., Rao H., Peng W., Yao H. (2019). Pharmacokinetics, Tissue Distribution, Metabolism, and Excretion of Naringin in Aged Rats. Front. Pharmacol..

[44] Zhang Y., Huo M., Zhou J., Xie S. (2010). PKSolver: An add-in program for pharmacokinetic and pharmacodynamic data analysis in Microsoft Excel. Comput. Methods Programs Biomed..

[45] Chinese Pharmacopoeia Commission (2020). Guidance for Bioanalytical Method Validation, Pharmacopoeia of the People’s Republic of China.

